# G protein–coupled receptor kinase phosphorylation of distal C-tail sites specifies βarrestin1-mediated signaling by chemokine receptor CXCR4

**DOI:** 10.1016/j.jbc.2022.102351

**Published:** 2022-08-06

**Authors:** Ya Zhuo, Joseph M. Crecelius, Adriano Marchese

**Affiliations:** Department of Biochemistry, Medical College of Wisconsin, Milwaukee, Wisconsin, USA

**Keywords:** GPCR, phosphorylation, arrestin, GRK, BRET, 95% CI, 95% confidence interval, β_2_AR, β_2_-adrenergic receptor, βarr1, βarrestin1, AT_1a_, angiotensin II type 1A receptor, BRET, bioluminescence resonance energy transfer, BSA, bovine serum albumin, C-tail, carboxyl-terminal tail, CXCR, chemokine receptor, DMEM, Dulbecco's modified Eagle's medium, ERK1/2, extracellular signal–regulated kinase 1/2, FAK, focal adhesion kinase, FBS, fetal bovine serum, GPCR, G protein–coupled receptor, GRK, G protein–coupled receptor kinase, HA, hemagglutinin, HEK293, human embryonic kidney 293 cell line, MAP, mitogen-activated protein, NTS_1_, neurotensin receptor 1, PEI, polyethylenimine, Rluc, *Renilla* luciferase, Ser, serine, STAM, signal-transducing adaptor molecule, TBS, Tris-buffered saline, TBS-T, Tris-buffered saline with Tween-20, Thr, threonine

## Abstract

G protein–coupled receptor (GPCR) kinases (GRKs) and arrestins mediate GPCR desensitization, internalization, and signaling. The spatial pattern of GPCR phosphorylation is predicted to trigger these discrete GRK and arrestin-mediated functions. Here, we provide evidence that distal carboxyl-terminal tail (C-tail), but not proximal, phosphorylation of the chemokine receptor CXCR4 specifies βarrestin1 (βarr1)-dependent signaling. We demonstrate by pharmacologic inhibition of GRK2/3-mediated phosphorylation of the chemokine receptor CXCR4 coupled with site-directed mutagenesis and bioluminescence resonance energy transfer approaches that distal, not proximal, C-tail phosphorylation sites are required for recruitment of the adaptor protein STAM1 (signal-transducing adaptor molecule) to βarr1 and focal adhesion kinase phosphorylation but not extracellular signal–regulated kinase 1/2 phosphorylation. In addition, we show that GPCRs that have similarly positioned C-tail phosphoresidues are also able to recruit STAM1 to βarr1. However, although necessary for some GPCRs, we found that distal C-tail sites might not be sufficient to specify recruitment of STAM1 to βarr1 for other GPCRs. In conclusion, this study provides evidence that distal C-tail phosphorylation sites specify GRK–βarrestin-mediated signaling by CXCR4 and other GPCRs.

G protein–coupled receptor (GPCR) kinases (GRKs) and arrestins are important for regulating the duration and magnitude of GPCR signaling (1). GRKs phosphorylate agonist-activated GPCRs at serine (Ser) or threonine (Thr) residues, which trigger the recruitment and high-affinity binding of arrestins (2). Arrestin binding prevents heterotrimeric G protein coupling (3) and promotes receptor endocytosis *via* clathrin-coated pits (4). Arrestins also mediate G protein–independent signaling by functioning as ligand-regulated transducers and scaffolds of several signaling molecules (5, 6, 7). These functions of arrestins are generalizable to all GPCRs; however, these functions can diverge dependent on the ligand activating the same GPCR, and the dominant function of arrestin can vary between GPCRs (8). Although the mechanistic basis for this is beginning to emerge, it still remains poorly understood.

GRKs mediate agonist-promoted phosphorylation of GPCRs at Ser or Thr residues within the intracellular loops and/or carboxyl-terminal tail (C-tail) (9, 10). Individual GRKs phosphorylate discrete Ser or Thr residues culminating in discrete phosphorylation patterns (11). For example, upon agonist activation of the chemokine receptor CXCR4, GRK2/3 phosphorylates distal Ser/Thr sites on the C-tail, whereas GRK6 phosphorylates more membrane proximal Ser/Thr sites (12). Similarly, GRK2/3 phosphorylates distal Ser/Thr sites on the C-tail of the β_2_-adrenergic receptor (β_2_AR), whereas GRK6 phosphorylates proximal Ser/Thr sites (13). Importantly, specific and distinct patterns of phosphorylation produced by individual GRKs correlate with the divergent functions of β-arrestins (13). In this way, the phosphorylation pattern produced by an individual GRK encodes a specific signaling or functional outcome and has been referred to as a “barcode” (14).

Arrestins typically engage with phosphoresidues found within the C-tail of GPCRs, and they may also engage with phosphoresidues located on the intracellular loops (15). The number of phosphates and the spacing between them are particularly important to arrestin binding and activation (16, 17, 18, 19). The crystal structure of rhodopsin and arrestin-1 revealed that negatively charged phosphates may be accommodated by positively charged pockets on the surface of arrestin (19). Divergent phosphorylation patterns on GPCRs may be a factor in specifying discrete arrestin conformations that selectively expose regions where signaling molecules bind (20), culminating in different outcomes on β-arrestin-mediated signaling. While phosphorylation patterns are beginning to explain activation of certain signaling pathways, the full scope on β-arrestin-mediated signaling remains poorly understood.

CXCL12 activates focal adhesion kinase (FAK) *via* balanced contributions from β-arrestin1-dependent (21, 22) and G protein-dependent pathways (23). We have previously shown that the β-arrestin1 pathway requires the adaptor protein STAM1 (signal-transducing adaptor molecule), which is a protein required for lysosomal trafficking and signaling of CXCR4 (24, 25, 26). Disruption of the interaction between βarr1 and STAM1 with minigenes from βarr1 or STAM1 (21) or overexpression of a βarr1 variant that shows reduced binding to STAM1 (22) selectively attenuates FAK, but not extracellular signal–regulated kinase 1/2 (ERK1/2), phosphorylation promoted by CXCL12. The interaction between βarr1 and STAM1 and phosphorylation of FAK are required for chemotaxis toward CXCL12 in HeLa cells (21, 22). STAM1 is also required for CXCR4-mediated phosphorylation of ERK1/2 and Akt, although this does not require β-arrestins (25, 26). The STAM1-binding site maps to the base of the finger loop on βarr1, distinct from where other non–GPCR-binding partners bind, although it partially overlaps with the GPCR-binding site (22). Binding to this novel surface on βarr1 may explain why STAM1 specifies FAK signaling by CXCR4, but how STAM1 engagement with βarr1 results in FAK activation remains to be determined.

In this study, we addressed the receptor determinants specifying STAM1 recruitment to βarr1 following CXCR4 activation and the broad applicability to other GPCRs. Here, we provide evidence that ligand-stimulated phosphorylation of the distal C-tail of CXCR4 is required for βarr1-mediated signaling by STAM1. We show that CXCL12-stimulated phosphorylation of FAK, but not ERK1/2, requires GRK2/3-promoted phosphorylation of phosphosites located at the distal C-tail of CXCR4 but not membrane proximal phosphosites. Importantly, the distal phosphosites are required to recruit the adapter protein STAM1 to βarr1. This information is transposable because swapping the C-tail of a GPCR that does not recruit STAM1 to βarr1 with the C-tail of CXCR4 is sufficient to confer the ability of this GPCR to recruit STAM1 to βarr1, indicating the information dictating this interaction resides solely within the C-tail. We found that other GPCRs, but not all, that contain potential phospho-Ser or phospho-Thr residues at their distal C-tail are also able to recruit STAM1 to βarr1. Our data suggest that distal, but not the proximal, C-tail phosphosites on CXCR4 and other GPCRs facilitates STAM1 recruitment to βarr1 and FAK activation.

## Results

### Site-specific phosphorylation of CXCR4 specifies FAK phosphorylation

**Figure 1:**
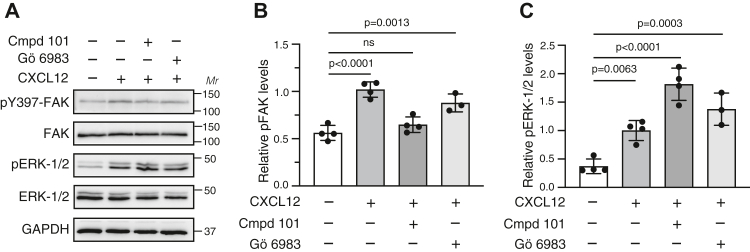
**GRK2/3 inhibitor reduces CXCL12-stimulated FAK phosphorylation.***A*, HeLa cells were stimulated with 50 nM CXCL12 for 15 min at 37 °C in the presence of either vehicle, 30 μM GRK2/3 inhibitor (compound 101) or 1 μM PKC inhibitor (Gö6983). Whole-cell lysates were analyzed by immunoblotting for phosphorylated FAK or ERK1/2 and total FAK or ERK1/2. Representative immunoblots from four independent experiments are shown. Bars represent the mean ± SD. values of pY397-FAK (*B*) or pERK1/2 (*C*) normalized to FAK or ERK1/2 relative to the signal from cells treated with CXCL12 without either kinase inhibitor as determined by densitometry-based quantification. Data were analyzed by one-way ANOVA followed by Bonferroni’s multiple comparison test. *p* Values between indicated groups are shown, and *p* < 0.05 was considered statistically significant. ERK1/2, extracellular signal–regulated kinase 1/2; FAK, focal adhesion kinase; GRK, G protein–coupled receptor kinase.

To better understand CXCR4 signaling, we sought to identify the kinases and receptor determinants responsible for CXCR4-promoted FAK activation. We used HeLa cells, which express CXCR4 endogenously (12, 27) and which we have previously used to study CXCR4 trafficking and signaling (20, 21, 22, 24, 25, 28, 29, 30, 31). CXCL12, the cognate ligand for CXCR4, also binds to the atypical chemokine receptor ACKR3, which is also expressed endogenously in HeLa cells (32) and is mostly recognized as a nonsignaling scavenging receptor for CXCL12 (33). HeLa cells devoid of ACKR3, but not CXCR4, signal normally in response to CXCL12 stimulation (33), which is consistent with our previous reports suggesting that CXCL12 signaling in HeLa cells is mediated exclusively by CXCR4 (20, 30). The C-tail of CXCR4 contains multiple Ser and Thr residues that are phosphorylated by GRK and PKC isoforms (12, 27). We treated HeLa cells with 30 μM of compound 101, a selective GRK2/3 inhibitor (34) or 1 μM of Gö6983, a broad spectrum PKC inhibitor (35), and examined FAK and ERK1/2 phosphorylation by immunoblotting with phospho-specific antibodies after stimulation with CXCL12. Treatment with compound 101 reduced FAK phosphorylation by CXCL12, whereas the PKC inhibitor had minimal effect (Fig. 1, *A* and *B*). In contrast, CXCL12 promoted that ERK1/2 phosphorylation was elevated by treatment with compound 101 but not treatment with Gö6983 (Fig. 1, *A* and *C*). These data suggest that GRK2/3 phosphorylation of CXCR4, but not PKC, is required for FAK signaling promoted by CXCL12, whereas GRK2/3 suppresses ERK1/2 signaling. This effect on ERK1/2 signaling is similar to what has been previously observed with RNAi against GRK2 (12). It remains unclear why GRK2/3 negatively regulates ERK1/2 phosphorylation by CXCR4, but it may be because GRK2 directly impacts the upstream mitogen-activated protein (MAP) kinase kinase that phosphorylates ERK1/2 (36).

**Figure 2:**
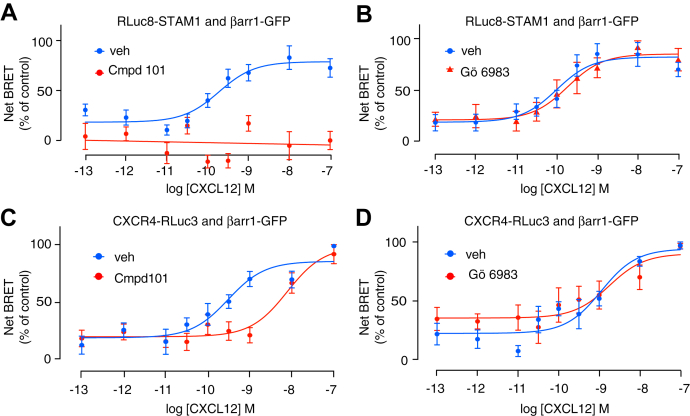
**GRK2/3 inhibitor reduces STAM1 recruitment to βarr1 in the presence of increasing concentrations of CXCL12.***A* and *B*, HEK293 cells transiently coexpressing HA-CXCR4, RLuc8-STAM1, and βarr1-GFP were stimulated at 37 °C with increasing concentrations of CXCL12 in the presence of either vehicle, 30 μM GRK2/3 inhibitor (compound 101) (*A*) or 1 μM PKC inhibitor (Gö6983) (*B*). BRET was measured at room temperature 10 min after the addition of CXCL12. Data are the mean ± SEM. BRET values normalized to the maximal response from six (*A*) or five (*B*) independent experiments performed in triplicate. *C* and *D*, HEK293 cells transiently coexpressing CXCR4-RLuc3 and βarr1-GFP were stimulated with increasing concentrations of CXCL12 in the presence of either vehicle, 30 μM compound 101 (*C*), or 1 μM Gö6983 (*D*) at 37 °C, which was followed by BRET measurements at room temperature. Data shown are the mean ± SEM. BRET values normalized to the maximal response from six independent experiments performed in triplicate. All curves were fit by nonlinear regression based on a single binding site model or linear regression using GraphPad Prism. βarr1, βarrestin1; BRET, bioluminescence resonance energy transfer; GRK, G protein–coupled receptor kinase; HA, hemagglutinin; HEK293, human embryonic kidney 293 cell line; STAM1, signal-transducing adaptor molecule 1.

Previously, we reported that FAK phosphorylation by CXCL12 stimulation of CXCR4 is mediated by a complex formed by βarr1 and STAM1 (21, 22). To study the βarr1 and STAM1 interaction, we recently used bioluminescence resonance energy transfer (BRET) in human embryonic kidney 293 (HEK293) cells heterologously expressing hemagglutinin (HA)-tagged CXCR4 with donor *Renilla* luciferase (Rluc)–tagged STAM1 and acceptor GFP-tagged βarr1 (βarr1-GFP) treated with varying doses of CXCL12 (22). We previously used STAM1 tagged with Rluc at the C terminus (22), whereas here to examine whether the GRK2/3 or PKC inhibitor impacts the interaction between βarr1 and STAM1 by BRET, we used STAM1 tagged at the N terminus with Rluc8 (37). Stimulation of cells with CXCL12 resulted in a dose-dependent increase in BRET between Rluc8-STAM1 and βarr1-GFP (Fig. 2*A*), in agreement with our previous report indicating that the interaction between βarr1 and STAM1 is promoted by stimulation of CXCR4 (22). The GRK2/3 inhibitor completely blocked the BRET response (Fig. 2*A*), whereas the PKC inhibitor had no effect on the BRET response (Fig. 2*B*). We also examined the effect of the GRK2/3 or PKC inhibitor by BRET on the recruitment of βarr1-GFP to Rluc3-tagged CXCR4 in HEK293 cells following stimulation with CXCL12. CXCL12 promoted a dose-dependent increase in BRET between βarr1-GFP and CXCR4-Rluc3 that was shifted to the right in the presence of the GRK2/3 inhibitor (Fig. 2*C*). The EC_50_ values in the presence of vehicle or GRK2/3 inhibitor are 0.30 nM (95% confidence interval [95% CI] = 0.11–0.79) and 7.79 nM (95% CI = 2.89–20.31), respectively. In contrast, the PKC inhibitor had little effect on the BRET response (Fig. 2*D*). These data suggest that βarr1 recruitment to CXCR4 and STAM1 recruitment to βarr1 requires GRK2/3 phosphorylation of CXCR4 but not phosphorylation by PKC.

**Figure 3:**
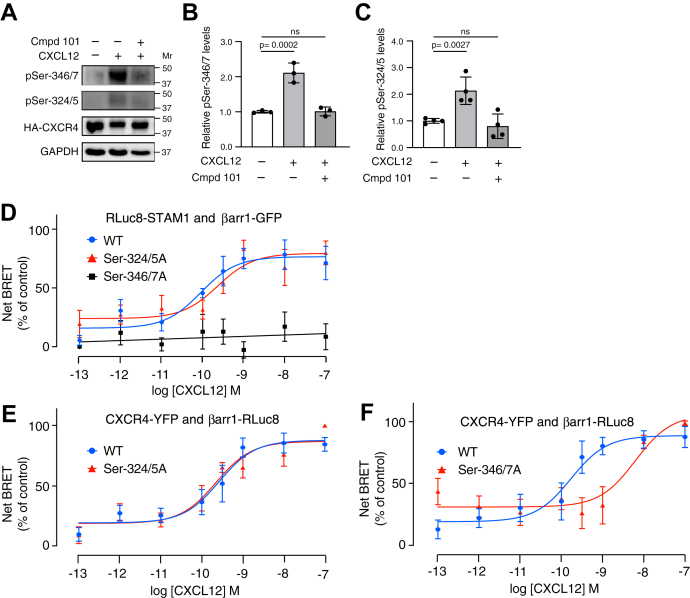
**Phosphorylation of CXCR4 at Ser-346/7 is required for STAM1 recruitment to βarr1 following CXCL12 stimulation.***A*, HEK293 cells transiently expressing HA-CXCR4 were pretreated with either vehicle or 30 μM GRK2/3 inhibitor (compound 101) followed by stimulation with 50 nM CXCL12 for 15 min at 37 °C. Cleared cell lysates were analyzed by immunoblotting to detect indicated phosphorylated residues on CXCR4. Representative immunoblots are shown. Bars represent the mean ± SD of pSer-346/7 (*B*) or pSer-324/5 (*C*) normalized to HA-CXCR4 relative to the signal from cells without stimulation and inhibitor treatment as determined by densitometry-based quantification from three (*B*) or four (*C*) independent experiments. Data were analyzed by one-way ANOVA followed by Bonferroni’s multiple comparison test. *p* Values between indicated groups are shown, and *p* < 0.05 was considered statistically significant. *D*, HEK293 cells transiently coexpressing with either HA-tagged WT CXCR4, Ser-324/5A or Ser-346/7A plus RLuc8-STAM1, and βarr1-GFP were stimulated at 37 °C with increasing concentrations of CXCL12 for 10 min before BRET measurements. Data shown are the mean ± SEM. BRET values normalized to the maximal response from six independent experiments performed in triplicate. The relative cell surface levels of HA-tagged Ser346/7A (1.01 ± 0.05) and Ser324/5A (1.28 ± 0.15) were similar to HA-CXCR4 (1 ± 0.22). *E* and *F*, HEK293 cells transiently coexpressing either WT CXCR4-YFP, Ser-324/5A-YFP or Ser-346/7A-YFP, and βarr1-RLuc8 were stimulated at 37 °C with increasing concentrations of CXCL12 followed by BRET measurements. Data shown are mean BRET ± SEM values normalized to the maximal response from six independent experiments performed in triplicate. All curves were fit by nonlinear regression based on a single binding site model or simple linear regression using GraphPad Prism. βarr1, βarrestin1; BRET, bioluminescence resonance energy transfer; HA, hemagglutinin; HEK293, human embryonic kidney 293 cell line; Ser, serine; STAM1, signal-transducing adaptor molecule 1.

To examine which phosphorylated residues are required for STAM1 recruitment to βarr1, we examined CXCR4 phosphorylation using site-specific antiphospho antibodies. CXCL12 stimulation promotes phosphorylation of several C-tail Ser and Thr residues, and here, we focused on Ser pairs Ser-346/Ser-347 (Ser-346/7), located at the distal end of the C-tail, and Ser-324/Ser-325 (Ser-324/5), located more membrane proximal on the C-tail (12). We used commercially available anti-pSer-346/7 and anti-pSer-324/5 antibodies to detect CXCL12-stimulated phosphorylation in CXCR4-transfected HEK293 cells, which have been used extensively to study CXCR4 phosphorylation and signaling (12). As we and others have previously shown (12, 38), CXCL12 stimulation promotes phosphorylation at both Ser-346/7 and Ser-324/5 (Fig. 3*A*). The GRK2/3 inhibitor significantly reduced phosphorylation at Ser-346/7 (Fig. 3*B*) or Ser-324/5 (Fig. 3*C*) upon CXCL12 stimulation. Previous studies have shown that CXCR4 phosphorylation is hierarchical in nature whereby phosphorylation of Ser-324/5 is dependent upon initial phosphorylation at Ser-346/7 by GKR2/3 (39), which might explain why the GRK2/3 inhibitor reduced phosphorylation at Ser-324/5, even though this Ser pair is phosphorylated by GRK6 (12).

We next examined whether STAM1 is recruited to βarr1 by BRET in HEK293 cells expressing CXCR4 phospho-deficient receptors in which Ser-324/5 or Ser-346/7 are substituted to Ala residues (Ser-324/5A or Ser-346/7A). There was a dose-dependent BRET response between Rluc8-STAM1 and βarr1-GFP in cells expressing wildtype CXCR4 (Fig. 3*D*). The BRET response was effectively blocked in cells expressing Ser-346/7A but not in cells expressing Ser-324/5A (Fig. 3*D*). We also examined the BRET response between the variant receptors and βarr1. Previously, we have shown that CXCR4-YFP, but not variant Ser-324/5A-YFP, is robustly phosphorylated at Ser-324/5 (38); therefore, for these BRET experiments, the donor Rluc8 was tagged at the C terminus of βarr1 and the acceptor YFP was tagged at the C terminus of CXCR4 or receptor variants. The dose-dependent BRET response between βarr1-Rluc8 and Ser-324/5A-YFP was similar to the wildtype CXCR4-YFP (Fig. 3*E*), whereas the BRET response between βarr1-Rluc8 and Ser-346/7A-YFP was shifted to the right (Fig. 3*F*), suggesting reduced βarr1 recruitment to CXCR4. The relative surface expression of HA-CXCR4 (1.00 ± 0.22) to HA-Ser324/5A (1.28 ± 0.15) and HA-Ser-346/7A (1.01 ± 0.05) was similar as determined by ELISA. Taken together, these data suggest that phosphorylation of Ser-346/7 at the distal C-tail of CXCR4 promotes βarr1 recruitment to CXCR4, which we propose is necessary for STAM1 recruitment to βarr1.

**Figure 4:**
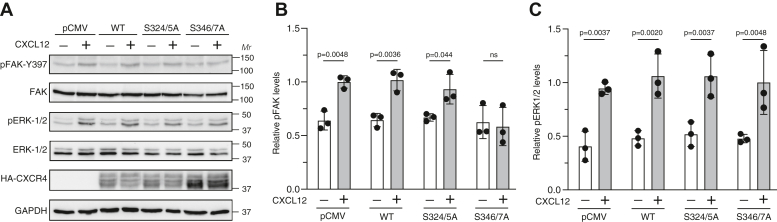
**Ser-346/7 pair is required for CXCL12-promoted FAK phosphorylation.***A*, HeLa cells transiently expressing either empty vector (pCMV) or HA-tagged WT CXCR4, S324/5A, or S346/7A were treated with either vehicle or 50 nM CXCL12 for 15 min. Whole-cell lysates were analyzed by immunoblotting for pY397-FAK or pERK1/2 and total FAK or ERK1/2. Representative immunoblots from four independent experiments are shown. *B* and *C*, bars represent the mean ± SD of pY397-FAK (*B*) or pERK1/2 (*C*) normalized to FAK or ERK1/2, respectively, relative to pCMV-transfected cells stimulated with CXCL12 as determined by densitometry-based quantification. Data were analyzed by one-way ANOVA followed by Bonferroni’s multiple comparison test. *p* Values between indicated groups are shown, and *p* < 0.05 was considered statistically significant. CMV, cytomegalovirus; FAK, focal adhesion kinase; HA, hemagglutinin; pERK1/2, phosphorylated extracellular signal–regulated kinase 1/2; Ser, serine.

Phosphorylation of Ser-324/5 by GRK6 is required for CXCR4 ubiquitination and lysosomal trafficking (31, 40), whereas phosphorylation of Ser-346/7 is mediated by GRK2 or GRK3 and is required for βarr1 or βarr2 recruitment leading to CXCR4 desensitization (27). However, the role of these phosphoresidues in promoting CXCR4 signaling remains poorly understood. Previously, we reported that transient expression of a βarr1 variant that shows reduced binding to STAM1 reduces CXCL12-mediated phosphorylation of FAK, but not ERK1/2, in HeLa cells (22). Here, we examined whether transient expression of wildtype CXCR4 or variants Ser-324/5A or Ser-346/7A in HeLa cells impacts FAK phosphorylation by CXCL12 stimulation. We observed CXCL12-stimulated FAK or ERK1/2 phosphorylation *via* endogenous CXCR4 in transiently transfected HeLa cells with empty vector (Fig. 4), similar to our previous findings (21). Phosphorylation of FAK or ERK1/2 was not enhanced in cells transfected with wildtype CXCR4 (Fig. 4), likely because endogenous CXCR4 signaling is limiting in HeLa cells. In contrast, FAK phosphorylation was reduced in cells transfected with Ser-346/7A (Fig. 4, *A* and *B*), possibly because of a dominant negative effect on endogenous CXCR4 signaling, similar to what has been observed in other cells when transfected with other CXCR4 variants (41). Although the mechanism of this dominant-negative effect remains to be clearly established, it is possible that CXCR4 might form into signaling competent nanoclusters (42), which could potentially be disrupted by the presence of the Ser-346/7 variant. Interestingly, transfection with Ser-324/5A did not impact FAK phosphorylation mediated by CXCL12 (Fig. 4, *A* and *B*). Importantly, CXCL12-stimulated ERK1/2 phosphorylation in Ser-324/5A or Ser-346/7A transfected cells was similar to wildtype receptor or empty vector (Fig. 4, *A* and *C*), indicating that CXCR4 signaling was not globally impacted.

### C-tail chimera between CXCR4 and CCR5 swaps the ability to recruit STAM1 to βarr1

**Figure 5:**
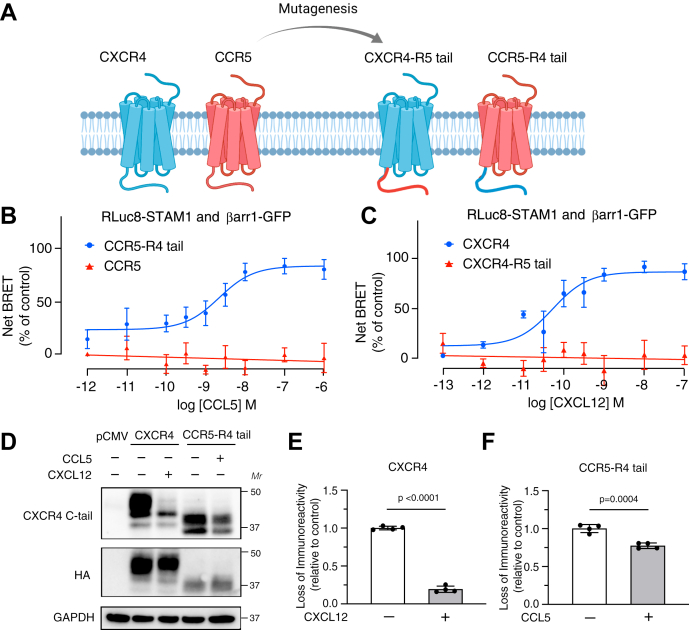
**CXCR4 and CCR5 C-tail swaps switches their ability to promote STAM1 recruitment to βarr1.***A*, schematic representation of the CXCR4 and CCR5 C-tail swapped chimeric receptors. HEK293 cells transiently coexpressing HA-tagged CCR5 or CCR5-R4 tail (*B*) and either HA-tagged CXCR4 or CXCR4-R5 tail (*C*) plus RLuc8-STAM1 and βarr1-GFP were stimulated with increasing concentrations of CCL5 (*B*) or CXCL12 (*C*). BRET measurements were carried out after 10 min of agonist stimulation at 37 °C. Data shown are mean ± SEM. BRET values normalized to the maximal response from six independent experiments performed in triplicate. The relative cell surface levels of HA-CCR5 (1.00 ± 0.18) to HA-CCR5 R4-tail (0.87 ± 0.18) and HA-CXCR4 (1.00 ± 0.20) to HA-CXCR4 R5-tail (1.38 ± 0.24) were similar. All curves were fit by nonlinear regression based on a single binding site model using GraphPad Prism. *D*, HEK293 cells transiently expressing either pCMV, HA-CXCR4, or HA-CCR5-R4 tail were stimulated by either 50 nM CXCL12 or 500 nM CCL5 for 15 min. Cleared cell lysates were analyzed by immunoblotting with an antibody that recognizes the unphosphorylated C-tail of CXCR4. Loss of immunoreactivity reflects increased phosphorylation of the receptor C-tail. Representative immunoblots are shown from four independent experiments. *E* and *F*, bar graphs represent the mean ± SD loss of immunoreactivity normalized to HA immunoblot relative to vehicle-treated cells as determined by densitometry-based quantification. Data were analyzed by unpaired *t* test. *p* Values between indicated groups are shown, and *p* < 0.05 was considered statistically significant. βarr1, βarrestin1; BRET, bioluminescence resonance energy transfer; CMV, cytomegalovirus; HA, hemagglutinin; HEK293, human embryonic kidney 293 cell line; STAM1, signal-transducing adaptor molecule 1.

Our data suggest that the position of the phosphoresidues at the distal C-tail of CXCR4 is necessary to promote the interaction between βarr1 and STAM1. The chemokine receptor CCR5 has been shown to be phosphorylated and to recruit βarr1 following agonist activation (43, 44); however, it does not have Ser or Thr clusters at the distal C-tail, suggesting that this GPCR may not be able to recruit STAM1 to βarr1. To examine this, we swapped the C-tails between CXCR4 and CCR5 and examined the ability of the chimeric receptors to promote the interaction between βarr1 and STAM1 by BRET (Fig. 5*A*). There was no dose-dependent BRET response between Rluc8-STAM1 and βarr1-GFP in HEK293 cells transiently expressing FLAG-CCR5 treated with CCL5, the cognate ligand for CCR5 (Fig. 5*B*). However, in HEK293 cells transiently expressing chimeric receptor CCR5 with the C-tail of CXCR4 (CCR5-R4 tail), CCL5 stimulation dose-dependently increased the BRET response between Rluc8-STAM1 and βarr1-GFP (Fig. 5*B*). In contrast, in HEK293 cells transiently expressing chimeric receptor CXCR4 with the C-tail of CCR5 (CXCR4-R5 tail), there was no BRET response between Rluc8-STAM1 and βarr1-GFP following dose-dependent stimulation with CXCL12 when compared with CXCR4 (Fig. 5*C*). The CCR5-R4 tail chimera was phosphorylated following stimulation with CCL5, as determined by loss of immunoreactivity of an antibody that specifically recognizes an epitope of the unphosphorylated C-tail of CXCR4 that includes Ser-346/7 (Fig. 5, *D*–*F*). The relative surface expression of HA-CCR5 (1.00 ± 0.18) to HA-CCR5-R4 tail (0.87 ± 0.18) and HA-CXCR4 (1.00 ± 0.05) to CXCR4-R5 tail (1.38 ± 0.24) was similar as determined by ELISA. These data indicate that the C-tail of CXCR4 is necessary and sufficient for STAM1 recruitment to βarr1, likely reflected by the presence of the distal C-tail Ser/Thr phosphoclusters.

**Figure 6:**
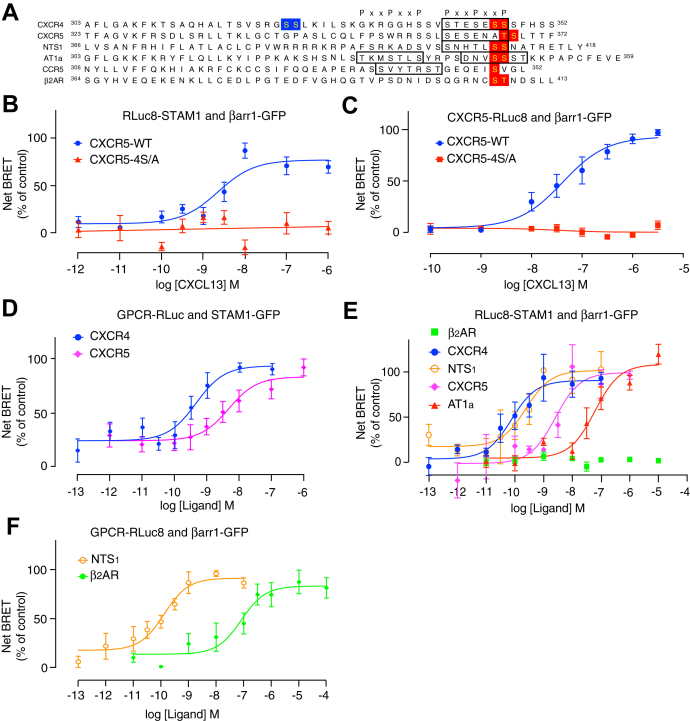
**Analysis of STAM1 recruitment to βarr1 by agonist activation of various GPCRs.***A*, amino acid sequence alignment of the carboxyl-terminal tails from CXCR4, CXCR5, NTS_1_, AT_1a_, CCR5, and β_2_AR. For CXCR4, Ser-324/5 are highlighted in *blue* and Ser-346/7 are highlighted in *red*. For the other GPCRs, Ser/Thr residues that best align with Ser-346/7 in CXCR4 are highlighted in *red*. The *boxed regions* represent the phospho-motif PxxPxxP, where P represents a phosphorylated Ser or Thr residue, or negatively charged amino acid, and x denotes any amino acid. *B*, HEK293 cells transiently coexpressing either FLAG-tagged CXCR5-WT or CXCR5-4S/A plus RLuc8-STAM1 and βarr1-GFP were stimulated with increasing concentrations of CXCL13 for 10 min at 37 °C followed by BRET measurements. Data shown are the mean ± SEM. BRET values normalized to the maximal response from six independent experiments performed in triplicate. The relative cell surface levels of HA-CXCR5 (1.00 ± 0.22) were similar to HA-CXCR5-4S/A (0.89 ± 0.21). *C*, HEK293 cells transiently coexpressing either WT CXCR5 or CXCR5-4S/A tagged with Rluc8 at the C terminus and βarr1-GFP were stimulated with increasing concentrations of CXCL13 followed by BRET measurements. Data shown are the mean ± SEM. BRET values normalized to the maximal response from three independent experiments performed in triplicate. *D*, HEK293 cells transiently coexpressing either CXCR4-Rluc3 or CXCR5-Rluc8 with STAM1-GFP were stimulated for 10 min at 37 °C with increasing concentrations of CXCL12 or CXCL13, respectively, before BRET measurements. Data shown are the mean ± SEM. BRET values normalized to the maximal response from five independent experiments performed in triplicate. *E*, HEK293 cells transiently coexpressing either FLAG-tagged CXCR4, CXCR5, NTS_1_, β_2_AR, or AT_1a_ plus RLuc8-STAM1 and βarr1-GFP were stimulated for 10 min at 37 °C with increasing concentrations of CXCL12, CXCL13, neurotensin, isoproterenol, or angiotensin II, respectively, followed by BRET measurements. Data shown are the mean ± SEM. BRET values normalized to the maximal response from four independent experiments performed in triplicate. The relative cell surface levels of FLAG-β_2_AR (0.69 ± 0.18) or FLAG-AT_1a_ (0.68 ± 0.06) to FLAG-CXCR4 (1.00 ± 0.05) were similar, whereas FLAG-NTS_1_ (2.37 ± 0.34) was somewhat higher. *F*, HEK293 cells transiently coexpressing either NTS_1_-RLuc8 or β_2_AR-RLuc8 and βarr1-GFP were stimulated with increasing concentrations of neurotensin or isoproterenol, respectively, for 10 min at 37 °C followed by BRET measurements. Data shown are the mean ± SEM. BRET values normalized to the maximal response from four independent experiments performed in triplicate. All curves were fit by nonlinear regression based on a single binding site model using GraphPad Prism. β_2_AR, β_2_-adrenergic receptor; βarr1, βarrestin1; AT_1a,_ angiotensin II type 1A receptor; BRET, bioluminescence resonance energy transfer; GPCR, G protein–coupled receptor; HA, hemagglutinin; HEK293, human embryonic kidney 293 cell line; NTS_1,_ neurotensin receptor 1; Ser, serine; STAM1, signal-transducing adaptor molecule 1.

To determine whether this is generalizable to other GPCRs, we examined a GPCR that contains potential phosphorylation sites at their distal C-tail. The chemokine receptor CXCR5, whose cognate ligand is CXCL13, has Ser/Thr phosphoclusters at the analogous position to CXCR4 in its C-tail (Fig. 6*A*). We examined the ability of agonist stimulation of CXCR5 to promote recruitment of STAM1 to βarr1 by BRET. In HEK293 cells transiently expressing FLAG-CXCR5, CXCL13 dose-dependently increased the BRET response between Rluc8-STAM1 and βarr1-GFP, consistent with agonist-dependent recruitment of STAM1 to βarr1 (Fig. 6*B*). In addition, CXCL13 dose-dependently increased the BRET response between CXCR5-Rluc8 and βarr1-GFP, consistent with agonist-dependent βarr1 recruitment to CXCR5 (Fig. 6*C*). Substitution of the distal Ser residues to Ala residues (CXCR5-4S/A) (Fig. 6*A*) completely blocked the BRET response between Rluc8-STAM1 and βarr1-GFP (Fig. 6*B*) and between βarr1-GFP and CXCR5-4S/A-Rluc8 (Fig. 6*C*). The relative cell surface expression of HA-CXCR5 (1.00 ± 0.22) to HA-CXCR5-4S/A (0.89 ± 0.21) was similar as determined by ELISA. Because STAM1 is likely recruited to βarr1 when it is bound to the C-tail of the GPCR, we examined whether we could observe BRET between CXCR4 or CXCR5 and STAM1. We detected ligand-induced BRET between CXCR4-RLuc3 and STAM1-GFP and between CXCR5-Rluc8 and STAM1-GFP in HEK293 cells cotransfected with βarr1 (Fig. 6*D*), suggesting that STAM1 and βarr1 form a complex with the agonist-activated GPCR.

We next examined whether other GPCRs are able to recruit STAM1 to βarr1. The neurotensin receptor 1 (NTS_1_), angiotensin II type 1A receptor (AT_1a_), and β_2_AR have Ser/Thr residues at the distal C-tail position, similar to CXCR4 and CXCR5 (Fig. 6*A*). We performed BRET experiments with each FLAG-tagged GPCR transiently coexpressed in HEK293 cells with Rluc8-STAM1 and βarr1-GFP. Neurotensin stimulation of NTS_1_ promoted a dose-dependent increase in the BRET response between βarr1-GFP and Rluc8-STAM1 (Fig. 6*E*), consistent with STAM1 recruitment to βarr1. The potency (0.22 nM, 95% CI = 0.06–0.69) was similar to the potency of βarr1-GFP recruitment to NTS_1_-Rluc8 (0.13 nM, 95% CI = 0.05–0.30) as determined by BRET (Fig. 6*F*). Angiotensin II stimulation of FLAG-AT_1a_ also promoted a dose-dependent BRET response between βarr1-GFP and Rluc8-STAM1 with a potency of 0.07 μM (95% CI = 0.02–0.20), although above the potency for βarr1 recruitment to AT_1a_ as measured by BRET (45), suggesting that AT_1a_ promotes a weak interaction between STAM1 and βarr1. In contrast, there was no isoproterenol-dependent BRET response between βarr1-GFP and Rluc8-STAM1 in cells transiently expressing FLAG-β_2_AR (Fig. 6*E*), despite the fact that isoproterenol stimulation promotes a BRET response between β_2_AR-Rluc8 and βarr1-GFP (Fig. 6*F*). The relative cell surface expression of the receptors FLAG-β_2_AR (0.69 ± 0.18), FLAG-AT1a (0.68 ± 0.06), and FLAG-NTS_1_ (2.37 ± 0.34) were similar to FLAG-CXCR4 (1.00 ± 0.05) as determined by ELISA. These data suggest that at least for some GPCRs, distal phospho-sites along the C-tail are not sufficient to specify STAM1 recruitment to βarr1, although necessary for others.

## Discussion

β-arrestins prefer to bind to agonist-activated and phosphorylated GPCRs, which sterically prevents heterotrimeric G protein binding and target GPCRs for endocytosis *via* clathrin-coated pits (46). In addition, β-arrestins instigate signaling pathways downstream of active GPCRs by serving as scaffolds for signaling molecules, such as MAP kinases (6, 47, 48, 49, 50, 51, 52, 53). The molecular basis for β-arrestin signaling, especially to signaling pathways other than MAP kinases, remains poorly understood. Here, we provide evidence that phosphorylation of Ser residues by GRK2/3 at the distal C-tail of CXCR4 is necessary to specify discrete βarr1-mediated signaling *via* recruitment of adaptor protein STAM1, a step that is necessary for activation of FAK but not activation of ERK1/2. This information is transposable because swapping the C-tail of a GPCR that does not recruit STAM1 to βarr1 with the C-tail of CXCR4 is sufficient to confer the ability of this GPCR to recruit STAM1 to βarr1, further suggesting that the information dictating this interaction resides solely within the C-tail. A related chemokine receptor, CXCR5, and the neurotensin receptor NTS_1_, similar to CXCR4, have analogous phosphoresidues at the distal C-tail, also recruit STAM1 to βarr1. However, CCR5 and β_2_AR are unable to recruit STAM1 to βarr1, although they too have phosphoresidues at the distal C-tail, suggesting that phosphoresidues alone at the distal C-tail are not sufficient to specify STAM1 recruitment to βarr1 and that other determinants may be important. Therefore, phosphorylation sites at the distal C-tail of some GPCRs, but not all, specifies βarr1-dependent signaling to FAK *via* the adaptor protein STAM1.

A key finding of our study is that GRK2/3 specifies discrete CXCR4 signaling by site-specific phosphorylation of distal C-tail site Ser-346/7. CXCL12-promoted phosphorylation at Ser-346/7 specifies FAK, not ERK1/2, phosphorylation (Fig. 4). In contrast, Ser-324/5 phosphorylation is not required for FAK or ERK1/2 phosphorylation (Fig. 4). These data are consistent with the barcode hypothesis, which proposes that distinct patterns of multisite phosphorylation encode GPCR signaling (13, 54, 55). While this has largely been addressed for GPCR-stimulated ERK1/2, C-Jun N-terminal kinase 3, or c-Src phosphorylation (56, 57, 58), here we extend this to include FAK phosphorylation. We have previously shown that phosphorylation of Ser-324/5 is required for ubiquitination and lysosomal degradation of CXCR4 (31, 38), further highlighting that site-specific phosphorylation of CXCR4 specifies discrete functional outcomes. CXCR4 phosphorylation on proximal Ser-324/5 has been linked to GRK6 (12). To our knowledge, phosphoresidues on CXCR4 that mediate ERK1/2 phosphorylation have yet to be defined, although phosphoresidues in other GPCRs have been linked to agonist-stimulated ERK1/2 phosphorylation (13, 56).

GRK-mediated phosphorylation of GPCRs is important for mediating high-affinity arrestin binding (18, 59). Previous studies have shown that βarr1 recruitment to CXCR4 by CXCL12 stimulation is mainly driven by phosphorylation of a distal Ser cluster within the C-tail that includes Ser-346/7 (12). This is consistent with our data that show that CXCL12-stimulated phosphorylation of Ser-346/7, but not Ser-324/5, efficiently promotes βarr1 recruitment to CXCR4 (Figs. 2*C* and 3, *E* and *F*). Remarkably, we show for the first time that phosphorylation of Ser-346/7 is necessary for STAM1 recruitment to βarr1 (Figs. 2*A* and 3*D*). Lack of STAM1 recruitment to βarr1 is consistent with the fact that GRK2/3-mediated phosphorylation of distal Ser-346/7 is also required for FAK phosphorylation (Figs. 1 and 4). Previously, we have shown that CXCR4-stimulated FAK phosphorylation, but not phosphorylation of ERK1/2, requires βarr1-mediated recruitment of the adaptor protein STAM1 (22). We also show that distal C-tail phosphosites on CXCR5 are also required for STAM1 recruitment to βarr1 (Fig. 6*B*), suggesting a common mechanism of FAK signaling by GPCRs. Because these sites on CXCR5 are also necessary for βarr1 recruitment (Fig. 6*B*), these data further suggest that STAM1 is recruited to receptor-bound βarr1. Consistent with this, we observed STAM1-GFP recruitment to CXCR4-Rluc3 or CXCR5-Rluc8 by BRET following CXCL12 or CXCL13 stimulation, respectively (Fig. 6*D*). Despite the fact that βarr1 is likely recruited to CXCR4 or CXCR5 at the plasma membrane, the location where STAM1 is recruited remains to be fully established. Previously, we have shown that βarr1 and STAM1 colocalize on early endosome antigen-1 (EEA1)–positive early endosomes with CXCR4 (24). We have also shown that βarr1, STAM1, and FAK colocalize at the cell periphery (21). This peripheral location would be consistent with the established role for the βarr1–STAM1 complex in receptor-mediated FAK phosphorylation and chemotaxis toward CXCL12 (21). However, the spatial and temporal relationship of the interaction between βarr1 and STAM1 and how GPCR phosphates specify this interaction remain to be investigated.

Our study also provides evidence for the first time that STAM1 recruitment to βarr1 is mediated by other GPCRs. GPCRs that have double Ser/Thr residues at their distal C-tail are able to recruit STAM1 to βarr1 (*e.g.*, CXCR5, NTS_1_, and AT_1a_) following agonist stimulation (Fig. 6, *A* and *D*). CCR5, which is unable to recruit STAM1 to βarr1 (Fig. 6*D*), has a single Ser residue at the analogous position to Ser-346/7 of CXCR4, suggesting that two phosphosites are required. However, the β_2_AR also has double Ser/Thr at the distal C-tail (Fig. 6*A*), and yet it is unable to recruit STAM1 to βarr1 (Figs. 5*B* and 6*D*). However, Ser-346/7 on CXCR4 are part of a larger phosphocluster of Ser/Thr residues and negative charged amino acids (Fig. 6*A*). Similarly, CXCR5, NTS1, and AT_1A_, but not CCR5 or β_2_AR, have large phosphoclusters in the analogous region of the distal C-tail (Fig. 6*A*). Therefore, it is possible that GPCRs with phosphoclusters at the distal C-tail may specify βarr1 binding and STAM1 recruitment, although additional GPCRs will have to be examined before we can make this generalization with confidence. It is important to note that the phosphorylation status of these GPCRs was not examined experimentally in this study. Recently, a predicted phosphorylation pattern (*e.g.*, *P*xx*P*xx*P*; *P* denotes phosphoresidue or can be a negatively charged residue, D/E) (Fig. 6*A*) was defined based on the crystal structure of rhodopsin in complex with arrestin-1 (19). The spacing of the negatively charged phosphates is predicted to fit into positively charged pockets on the surface of arrestin, which might explain βarrestin-mediated signaling (19). All the GPCRs examined in this study including CCR5, but not β_2_AR (Fig. 6*A*), have this phosphorylation pattern at the distal C-tail; therefore, this phosphorylation pattern *per se* may not be driving STAM1 recruitment to βarr1, and other determinants and/or structural features are required. Furthermore, how the βarr1–STAM1 complex subsequently leads to activation of FAK remains to be investigated.

β-arrestin binding to agonist-activated and GRK-phosphorylated GPCRs leads to desensitization, internalization, and also arrestin-dependent signaling (52). These functions can diverge depending on the ligand activating the same GPCR, and the dominant function of arrestin can vary between GPCRs; however, the receptor determinants responsible for this remain poorly understood. Here, we provide evidence that agonist-stimulated GRK-mediated phosphorylation at the distal C-tail of CXCR4 specifies βarr1-dependent signaling *via* the adaptor protein STAM1. This may be generalizable to GPCRs that have phosphoclusters at their distal C-tails. Understanding how site-specific receptor phosphorylation is translated into discrete arrestin-mediated signaling pathways is important to understand the full scope and complexity of GPCR signaling.

## Experimental procedures

### Cell culture, antibodies, and reagents

HeLa cells were from American Type Culture Collection, and HEK293 cells were from Microbix. Cells were maintained in Dulbecco's modified Eagle's medium (DMEM) (catalog no.: D5796) (Sigma) supplemented with 10% fetal bovine serum (FBS) (Omega). Rabbit monoclonal anti-FAK (catalog no.: 12636-1-AP) and mouse monoclonal anti-GAPDH (catalog no.: 60004-lg) antibodies were from Proteintech. The rabbit polyclonal anti-pTyr-397-FAK antibody (catalog no.: 44-624G) was from Life Technologies. The rabbit monoclonal anti-ERK1/2 (catalog no.: 4695) and anti-pERK1/2 (catalog no.: 4370) antibodies were from Cell Signaling Technology. The mouse monoclonal anti-HA antibody (catalog no.: 901515) was from BioLegend. The rabbit polyclonal anti-pS346/7-CXCR4 (catalog no.: 7TM0071B), anti-pS324/5-CXCR4 (catalog no.: 7TM0071A), and anti-CXCR4 (catalog no.: 7TM0071N-IC) antibodies were from 7TM Antibodies. The mouse monoclonal anti-GAPDH antibody (catalog no.: ab9482) was from Abcam. The mouse monoclonal anti-FLAG antibody (catalog no.: F4049), neurotensin (catalog no.: N6383), and isoproterenol (catalog no.: I6504) were from Sigma–Aldrich. CXCL12, CXCL13, and CCL5 were from Protein Foundry. Compound 101 (catalog no.: HB2840) was from Hello Bio. PKC inhibitor Gö6983 (catalog no.: 133053-19-7), angiotensin II (catalog no.: 4474-91-3), and cilengitide (catalog no.: 5870) were from Tocris Bioscience. Coelenterazine H (catalog no.: 10111) and coelenterazine 400a (catalog no.: 10125) were from Biotium. Polyethylenimine (PEI; catalog no.: 23966) was from Polysciences, Inc.

### DNA plasmids

The CXCR4-Rluc3 and βarrestin1-GFP^10^ plasmids were kind gifts from Nikolaus Heveker (Hôpital Sainte-Justine) and Michel Bouvier (Department de biochemie, Université de Montréal), respectively. Rluc3 was previously described by Leduc *et al.* (60) and is also known as RlucII (61). The HA-CXCR4, HA-CCR5, and chimeric HA-CXCR4-CCR5-C-tail and HA-CCR5-CXCR4-C-tail variant plasmids were described previously (40). The Rluc8-STAM1 plasmid was made using NEBuilder HiFi DNA assembly (New England BioLabs; catalog no.: E5520s) with an Rluc8 fragment amplified by PCR from pTRE-Tight-Rluc8 plasmid (pTRE-Tight-Rluc8 was a gift from Vladislav Verkhusha; Addgene plasmid #79844) and a PCR-amplified fragment of the pcDNA3 backbone containing the STAM1-coding region. The βarr1-Rluc8 plasmid was generated using NEBuilder HiFi DNA assembly with an Rluc8 fragment amplified by PCR from pTRE-Tight-Rluc8 plasmid and a PCR-amplified fragment of the pcDNA3 backbone containing the βarr1-coding region. HA-CXCR4-YFP, HA-CXCR4-S324/5A, and HA-CXCR4-YFP-S324/5A plasmids were described previously (29, 31). HA-CXCR4-S346/7A and HA-CXCR4-YFP-S346/7A plasmids were generated by QuickChange site-directed mutagenesis of HA-CXCR4 or HA-CXCR4-YFP using complimentary mutagenic primers and Platinum SuperFi DNA polymerase (Invitrogen; catalog no.: 12351). FLAG-CXCR4, FLAG-AT_1a_, and FLAG-β_2_AR were described previously (62). FLAG-CXCR5 was made using NEBuilder HiFi DNA assembly with a fragment of the FLAG containing backbone amplified by PCR from the FLAG-CXCR4 plasmid and a fragment of the CXCR5-coding region amplified by PCR from 3xHA-CXCR5 plasmid (cDNA Resource Center; catalog no.: CXCR50TN00). The FLAG-NTS_1_ plasmid was made by PCR using Platinum SuperFi DNA polymerase from the NTS_1_-Tango plasmid (NTS_1_-Tango was a gift from Bryan Roth; Addgene plasmid #66457) by removing the modules for the tobacco etch virus protease cleavage site, the tetracycline transactivator protein, and the vasopressin V2 receptor tail and recircularization with the KLD enzyme mix (New England Biolabs; catalog no.: M0554s). The NTS_1_-Rluc8 plasmid was made using NEBuilder HiFi DNA assembly with a fragment of Rluc8, amplified by PCR from pTRE-Tight-Rluc8 plasmid and the NTS_1_-coding region, amplified by PCR from the NTS_1_-Tango plasmid. The β_2_AR-Rluc8 plasmid was made using NEBuilder HiFi DNA assembly with an Rluc8 fragment amplified by PCR from pTRE-Tight-Rluc8 plasmid and a fragment of the β_2_AR containing the pcDNA backbone amplified by PCR. The four most C-terminal putative phosphorylation sites on the C-tail of CXCR5 (Thr-367, Ser-368, Thr-370, and Thr-371) were substituted for alanine residues (FLAG-CXCR5-4S/A and CXCR5-RLuc8-4S/A) using nonoverlapping back-to-back mutagenic primers encoding the alanine substitutions were used to amplify template DNA encoding FLAG-CXCR5 or CXCR5-RLuc8 using Platinum SuperFi DNA polymerase and circularized with KLD enzyme mix. The new plasmids described in this study and primers used to make new plasmids are listed in Table S1 (see Supporting information). All plasmids were confirmed by dideoxy sequencing.

### BRET assay

To measure GPCR-stimulated STAM1 recruitment to βarr1 by BRET^2^, HEK293 cells grown on 6-cm or 10-cm dishes were cotransfected with GPCR (2–3 μg), Rluc8-STAM1 (200–400 ng), and βarr1-GFP^10^ (600 ng–1.2 μg) using PEI. To measure βarr1 recruitment to the GPCR by BRET^2^, HEK293 cells grown on 10-cm dishes were cotransfected with GPCR-Rluc8 (100 ng) and βarr1-GFP^10^ (300 ng). To measure βarr1 recruitment to CXCR4 or variant receptor by BRET^1^, HEK293 cells grown on 6-cm dishes were cotransfected with HA-CXCR4-YFP or variants HA-CXCR4-YFP-S324/5A or HA-CXCR4-YFP-S346/7A (30 ng) and βarr1-RLuc8 (5 ng). Twenty-four hours later, cells were seeded into 96-well white microplates at a density of 20,000 to 50,000 cells/well in DMEM containing 5% FBS. The next day, cells were washed with Dulbecco’s PBS supplemented with 20 mM Hepes, pH = 7.4 and then replaced with 80 μl of the same buffer. For inhibitor treatment, cells were pretreated with either 30 μM compound 101 for 30 min or 1 μM Gö6893 for 15 min before stimulation with increasing concentrations of ligands for 15 min at 37 °C. After incubation with ligands, 5 μM luciferase substrate coelenterazine 400A (DeepBlue C) or 50 μM luciferase substrate coelenterazine H was added for BRET^2^ or BRET^1^, respectively. BRET measurements were performed with a BioTek Cytation 5 cell imaging multimode reader or a BMG Labtech LUMIstar OMEGA microplate reader. For BRET^2^ readings, Rluc donor emission was detected at 440 ± 40 nm and acceptor GFP emission was detected at 515 nm ± 15 nm bandwidth. For BRET^1^ readings, Rluc donor emission was detected at 470 nm ± 15 nm and YFP acceptor emission was detected at 530 nm ± 15 nm. For each BRET experiment, RLuc plasmid–transfected cells were included as an internal background control. BRET ratios were calculated by dividing the acceptor emission light intensity by the donor emission light intensity, as we previously described (22), from three consecutive reads taken every 45 s, and the resulting BRET ratios were averaged. Net BRET was then calculated by subtracting background BRET from the raw BRET for each ligand dose. All BRET experiments were performed while cells were attached to white-walled clear-bottom 96-well plates. Prior to reading, white tape was adhered to the bottom of the plate. For all BRET experiments, parallel samples from the same pool of transfected cells were analyzed for cell surface expression of each GPCR (see “Receptor Surface Expression” section later).

### Detection of phosphorylated CXCR4

HEK293 cells grown on 10-cm dishes were transiently transfected with HA-CXCR4 using PEI. Twenty-four hours post-transfection, cells were seeded onto 6-well plates. The next day, cells were serum starved in DMEM supplemented with 20 mM Hepes for 3 h. Cells were then pretreated with 30 μM GRK2/3 inhibitor (compound 101) for 30 min, followed by stimulation with vehicle or 50 nM CXCL12 for 5 min or 15 min at 37 °C. For receptor variants, HEK293 cells grown on 6-cm dishes were transiently transfected with empty vector (pCMV10), HA-CXCR4, HA-CXCR4-S346/7A, HA-CXCR4-S324/5A, or HA-CCR5-R4 tail using PEI. Twenty-four hours post-transfection, cells were seeded onto 6-well plates. The next day, cells were serum starved in DMEM supplemented with 20 mM Hepes for 3 h. Cells were stimulated with vehicle, 50 nM CXCL12, or 500 nM CCL5 for 15 min at 37 °C. Cells were washed once with ice-cold PBS and lysed in 250 μl lysis buffer (50 mM Tris, 150 mM NaCl, 0.1% SDS, 0.5% sodium deoxycholate, and 1% Triton X-100, pH 7.5) containing protease inhibitors and phosphatase inhibitors (10 mM sodium fluoride, 10 mM sodium pyrophosphate, and 10 mM sodium orthovanadate). Samples were sonicated and cleared by centrifugation at 13,000*g* for 30 min at 4 °C. Protein concentration of the supernatants was determined (Thermo Fisher Scientific; catalog no.: 22660), and equal amounts were analyzed by 10% SDS-PAGE, transferred to nitrocellulose, and blocked for 1 h in Tris-buffered saline (TBS) containing 0.1% Tween-20 (TBS-T) and 5% milk (w/v). Blots were incubated overnight at 4 °C with primary antibodies against CXCR4 (nonphospho) and (phosphosensitive) pSer346/7 or pS324/5. Blots were washed with TBS-T and incubated with anti-rabbit horseradish peroxidase–conjugated secondary antibody for 1 h at room temperature. Blots were washed extensively with TBS-T and developed with Promega ECL solution (catalog no.: W1001) on a ChemiDoc imaging system (Bio-Rad). Data were analyzed using ImageJ software (National Institutes of Health). Phosphorylated (Fig. 3) or nonphosphorylated (Fig. 5) CXCR4 normalized to HA-CXCR4 or HA-CCR5-R4 tail is represented as a fraction relative to vehicle-treated cells.

### Detection of FAK or ERK1/2 phosphorylation

Agonist-induced FAK or ERK1/2 MAP kinase phosphorylation was performed as previously described (22). HeLa cells grown on 6-cm dishes were transiently transfected with empty vector (pcDNA3), HA-CXCR4, HA-CXCR4-S346/7A, or HA-CXCR4-S324/5A using PEI. The next day, equal number of cells were seeded onto 6-well plates. The next day, cells were serum starved in DMEM supplemented with 20 mM Hepes for 3 h. To study the effect of GRK2 or PKC on FAK or ERK1/2 phosphorylation by CXCL12 stimulation of CXCR4, cells were pretreated with 30 μM compound 101 for 30 min or 1 μM Gö3896 for 15 min, followed by stimulation with vehicle or 50 nM CXCL12 for 15 min at 37 °C. Cells were washed once with ice-cold PBS and lysed in 300 μl 2× sample buffer (8% SDS, 10% glycerol, 5% β-mercaptoethanol, 37.5 mM Tris, pH 6.5, and 0.003% bromophenol blue). Samples were sonicated, and equal amounts were analyzed by 10% SDS-PAGE and immunoblotting. pY397-FAK and pERK1/2 were detected by anti-pY397-FAK and anti-pERK1/2 antibodies, respectively. Total levels (phosphorylated and nonphosphorylated) of FAK and ERK1/2 were detected by anti-FAK and anti-ERK1/2 antibodies, respectively. Immunoblots were quantified using the ImageJ. The levels of pY397-FAK or pERK1/2 were normalized to total FAK or ERK1/2, respectively. Data represent the average of normalized pFAK or pERK1/2 as a fraction to the signal from cells treated with CXCL12 without either kinase inhibitor (Fig. 1) or transfected with pCMV (Fig. 4).

### Receptor surface expression

Surface expression of receptors was examined by whole-cell ELISA assay, as previously described (24). Briefly, HEK293 cells grown on 6-cm dishes transiently transfected with FLAG-tagged or HA-tagged GPCRs were seeded in DMEM containing 10% FBS onto 24-well plates coated with poly-l-lysine (Sigma; catalog no.: P1399). The next day, cells were rinsed once with ice-cold TBS buffer (20 mM Tris, pH 7.5, 150 mM NaCl) and immediately fixed with 3.7% formaldehyde (Sigma; catalog no.: F8775) for 15 min. After three washes with TBS, cells were incubated for 45 min at room temperature in TBS containing 1% bovine serum albumin (BSA) and then incubated with an anti-FLAG alkaline phosphatase–conjugated antibody at a dilution of 1:1000 in TBS–1% BSA for 1 h at room temperature. Cells were washed three times with TBS followed by incubation with developing solution until the appearance of a light yellow color. For detection of HA-tagged receptors, cells were incubated with an anti-HA antibody at a dilution of 1:1000 in TBS–1% BSA for 1 h at room temperature. Cells were then washed three times with TBS followed by an incubation with TBS containing 1% BSA for 45 min. Then, cells were incubated with an alkaline phosphatase–conjugated mouse antibody at a dilution of 1:5000 in TBS–1% BSA for 1 h at room temperature. Cells were washed three times with TBS followed by incubation with developing solution until the appearance of a light yellow color. Reactions were quenched by transferring 100 μl of this solution to a 96-well plate containing 100 μl of 0.4 M NaOH. The 96-well plate was read at 405 nm single end-point reading. The relative cell surface levels ± the standard deviation from multiple independent experiments performed in triplicate are reported in the relevant text and figure legends.

### Data analysis

Data were analyzed using GraphPad Prism, version 9.3.1 software (GraphPad Software, Inc). Densitometric analysis of immunoblots was performed using ImageJ. The results from three or four independent experiments were analyzed by one-way ANOVA with Bonferroni’s multiple comparison test (*p* < 0.05 was considered significant). Differences between the two groups were tested with a Student’s *t* test, and the differences between three or more groups were tested by one-way or two-way ANOVA. For BRET data analysis, data were averaged from at least four independent experiments and fit to the nonlinear regression curve. BRET data were transformed using GraphPad Prism by minimum–maximum normalization to preserve the original relationships based on the equation X′ = (X − Xmin)/(Xmax − Xmin), where X′ is the newly transformed value. The EC_50_ values before and after transformation were similar.

## Data availability

All data associated with this study are contained within the article.

## Supporting information

This article contains supporting information.

## Conflict of interest

The authors declare that they have no conflicts of interest with the contents of this article.
