# Supplementary material for: G protein–coupled receptor kinase phosphorylation of distal C-tail sites specifies βarrestin1-mediated signaling by chemokine receptor CXCR4
Source: J Biol Chem. 2022 Aug 6;298(9):102351. doi: 10.1016/j.jbc.2022.102351 (PMC9465349; doi:10.1016/j.jbc.2022.102351)
Supplement: Supplemental Table S1 [file mmc1.docx]

| **Table 1. Plasmid names and construct information for plasmids made in this study.** | | | |
| --- | --- | --- | --- |
| Plasmid name | Template and Backbone | Primer name | Primer sequence (5’-3’) |
|  | pTRE-Tight-Rluc8 | pcDNA-F1 | GAGCTCGGATCCACTAGTAACGGCCGC |
| **Rluc8-STAM1** |  | Rluc8-STAM-R1 | GATCGAAGGGATTGGTGGCAAAAAGA  GGGGATCCCAGCTGCTCGTTCTTCAGCACT  CTCTCCAC |
|  | T7-STAM1-pcDNA | STAM-F1 | CCTCTTTTTGCCACCAATCCCTTCGATC |
|  |  | STAM-pcDNA-R1 | GCGGCCGTTACTAGTGGATCCGAGC  TCCTATAGCAGAGCCTTCTGAGAATATGG |
| **HA-CXCR4-S346/7A** | HA-CXCR4 | S346A/S347A-Fwd | CCACTGAGTCTGAGGCTGCAAGTTTTCA  CTCCAGC |
| **HA-CXCR4-YFP-S346/7A** | HA-CXCR4-YFP | S346A/S347A-Rev | GCTGGAGTGAAAACTTGCAGCCTCAGAC  TCAGTGG |
|  | HA-CXCR5 | FLAG-TEF-CXCR5-For | CGATGATGACACCGAATTCAACTACCCG  CTAACGCTGG |
| **FLAG-CXCR5** |  | CXCR5-(pcDNA3)-Rev | GAATAGGGCCCTCTAGATTAGAACGTGG  TGAGAGAGGTGG |
|  | FLAG-CXCR4-pcDNA3 | pcDNA3-FLAG-Rev | GAATTCGGTGTCATCATCGTCCTTG |
|  |  | pcDNA3 (Vector)-For | TAATCTAGAGGGCCCTATTCTAGAGGG |
| **FLAG-NTSR1** | FLAG-NTSR1-Tango | NTSR1-Detango-Rev | GTTTAAACGGGCCCTCTAGACTCGAGCTA  GTACAGCGTTTCCCGAGTGGCGTTTGAAC |
|  |  | F detango Primer | CTCGAGTCTAGAGGGCCCGTTTAAAC |
| **NTSR1-Rluc8** |  | RLuc8-Fwd | GGAGGATCCTCCGGAGGATCCTCCAAGG  TGTACGACCCCGAGCAGAGGAAG |
|  | pTRE-Tight-Rluc8 | RLuc8-Rev | GTTTAAACGGGCCCTCTAGACTCGAGCTA  CTGCTCGTTCTTCAGCACTCTCTCCAC |
|  | FLAG-NTSR1-Tango | F detango Primer | CTCGAGTCTAGAGGGCCCGTTTAAAC |
|  |  | NTSR1-Rev | GGAGGATCCTCCGGAGGATCCTCCGTA  CAGCGTTTCCCGAGTGGCGTTTGAACTCAG |
|  | βarr1-APEX2 | Arr2_Linker_R | GGAGGATCCTCCGGAGGATCCTCCTCT  GTTGTTGAGCTGTGGAGAGCCGGTACC  ATCCTCCTCTTCC |
| **βarr1-Rluc8** |  | F- Primer | CTCGAGCATGCATCTAGAGGGCCCTATTC |
|  | pTRE-Tight-Rluc8 | RLuc8-Fwd | GGAGGATCCTCCGGAGGATCCTCCAAGG  TGTACGACCCCGAGCAGAGGAAG |
|  |  | Rluc8-R-β-arr1 | GAATAGGGCCCTCTAGATGCATGCTCGAG  CTACCTGCTCGTTCTTCAGCACTCTCTCCAC |
|  | pTRE-Tight-Rluc8 | RLuc8-Fwd | GGAGGATCCTCCGGAGGATCCTCCAAGG  TGTACGACCCCGAGCAGAGGAAG |
| **β_2_AR -Rluc8** |  | RLuc8-Rev | GTTTAAACGGGCCCTCTAGACTCGAGCTA  CTGCTCGTTCTTCAGCACTCTCTCCAC |
|  | FLAG- β_2_AR | ADRB2-Fwd | CTCGAGTCTAGAGGGCCCGTTTAAACAG  CGGCCGCGACTCTAGATCATAATC |
|  |  | ADRB2-Rev | GGAGGATCCTCCGGAGGATCCTCCCA  GCAGTGAGTCATTTGTACTACAATTCCTCC  CTTGTGA |
| **FLAG-CXCR5-4S/A** | FLAG-CXCR5 | pcDNA3 (Vector)-For | TAATCTAGAGGGCCCTATTCTAGAGGG |
| **CXCR5-Rluc8-4S/A** | CXCR5-Rluc8 | R5-D2MUT-OPEN-R | GAACGCGGCGAGAGCGGCGGCATTCTCTG |
